# ATAD2 overexpression links to enrichment of B-MYB-translational signatures and development of aggressive endometrial carcinoma

**DOI:** 10.18632/oncotarget.4955

**Published:** 2015-07-22

**Authors:** Camilla Krakstad, Ingvild L. Tangen, Erling A. Hoivik, Mari K. Halle, Anna Berg, Henrica M. Werner, Maria B. Ræder, Kanthida Kusonmano, June X. Zou, Anne M. Øyan, Ingunn Stefansson, Jone Trovik, Karl-Henning Kalland, Hong-Wu Chen, Helga B. Salvesen

**Affiliations:** ^1^ Centre for Cancer Biomarkers, Department of Clinical Science, University of Bergen, Bergen, Norway; ^2^ Department of Gynecology and Obstetrics, Haukeland University Hospital, Bergen, Norway; ^3^ Computational Biology Unit, University of Bergen, Bergen, Norway; ^4^ Department of Internal Medicine and Department of Biochemistry and Molecular Medicine, UC Davis Comprehensive Cancer Center, University of California, Davis, CA, USA; ^5^ Centre for Cancer Biomarkers, Department of Clinical Medicine, University of Bergen, Bergen, Norway; ^6^ Department of Microbiology, Haukeland University Hospital, Bergen, Norway; ^7^ Department of Pathology, Haukeland University Hospital, Bergen, Norway

**Keywords:** endometrial cancer, ATAD2, biomarker, molecular profiling

## Abstract

We have explored the potential for clinical implementation of ATAD2 as a biomarker for aggressive endometrial cancer by investigating to what extent immunohistochemical (IHC) staining for ATAD2 is feasible, reflects clinical phenotype and molecular subgroups of endometrial carcinomas. Increased expression of the *ATAD2* gene has been implicated in cancer development and progression in a number of tissues, but few studies have investigated ATAD2 expression using IHC. Here we show that high ATAD2 protein expression is significantly associated with established clinical-pathological variables for aggressive endometrial cancer, also in the subset of estrogen receptor α (ERα) positive tumors. Protein and mRNA expression of ATAD2 were highly correlated (*P* < 0.001), suggesting that IHC staining may represent a more clinically applicable measure of ATAD2 level in routinely collected formalin fixed paraffin embedded specimens. Gene expression alterations in samples with high ATAD2 expression revealed upregulation of several cancer-related genes (B-MYB, CDCs, E2Fs) and gene sets that previously have been linked to aggressive disease and potential for new targeting therapies. Our results support that IHC staining for ATAD2 may be a clinically applicable biomarker reflecting clinical phenotype and targetable alterations in endometrial carcinomas to be further explored in controlled clinical trials.

## INTRODUCTION

One of today’s most important clinical challenges is to develop clinically applicable and robust biomarkers to predict prognosis and to select patients for customized systemic treatments more likely to be beneficiary [1, 2]. Endometrial cancer is one of the most common female cancers in industrialized countries, with rising incidence-rate. In general, the overall prognosis is good, however in 15-20% of patients the disease recurs. In contrast to breast cancer, neither hormone receptor status, nor other biomarkers are currently implemented in clinical use. More robust biomarkers that independently can predict prognosis in order to improve treatment of endometrial cancer need to be developed [3].

One of the hallmarks of cancer is sustained proliferation. Altered gene expression of regulators of proliferation or reprogramming of distinct cellular pathways allows the cancer cell to bypass normal cell cycle control. The gene *ATAD2* (ATPase family, AAA domain containing 2; also listed as ANCCA, pro2000) is predominantly expressed in male germ cells, but becomes overexpressed in several cancer forms. It was initially identified as a target gene of the proto-oncogene AIB1 and as a transcriptional coactivator of ERα in breast cancer cells [4]. Further studies demonstrated that ATAD2 is overexpressed in several types of human cancers, including breast cancer, prostate cancer and lung cancer [4–8]. Moreover, its elevated transcripts were early included in a number of prognostic cancer-gene signatures that are linked to cancer development and progression such as increased risks of distant metastasis in breast cancer [9, 10] and disease recurrence in endometrial cancer [11].

The ATAD2 protein acts as a transcriptional co-regulator, both in hormone dependent gene transcription through its interactions with the hormone receptors ER and AR [12], and in transcriptional regulation by E2Fs and MYC through their direct interaction [6, 13]. In hormone dependent cancer cells *ATAD2* is an estrogen and androgen responsive gene and also a co-activator for full activity of ER and AR [4, 14]. The ATAD2 protein has also received attention due to its “drugable” composition. In addition to its N-terminal part that interacts with E2F and AR, ATAD2 consists of a bromodomain capable of binding to acetylated histones, and the AAA ATPase domain that possesses ATPase activity and mediates protein multimerization [4, 5, 13]. Both the bromodomain and the AAA ATPase domain could be targeted by small molecule inhibitors, and the recent chemical synthesis of the ATAD2 bromodomain could facilitate further studies of the function of the protein [15]. Combined with its apparent wide overexpression in cancers, this leaves ATAD2 as a promising target for therapy in several cancers and calls for further investigations of its expression in specific cancer types.

Few studies have investigated ATAD2 protein expression by IHC, mainly due to lack of commercially available specific antibodies. We here apply an antibody previously shown to specifically detect ATAD2 expression in breast cancer [7] to explore the potential for clinical implementation of ATAD2 as a biomarker for aggressive disease in endometrial cancer. We have investigated to what extent IHC staining for ATAD2 is feasible, reflects clinical phenotype and molecular subgroups of endometrial carcinomas. To shed light on the biological processes involved in cancers with high ATAD2 protein, gene expression analyses were performed in parallel. Our results support ATAD2 as a prognostic marker in endometrial cancer, and also imply that ATAD2 might be a promising predictive marker for specific treatment in the future.

## RESULTS

### Increased ATAD2 expression associates with more aggressive stages of disease and is correlated to copy-number alterations

**Figure 1:**
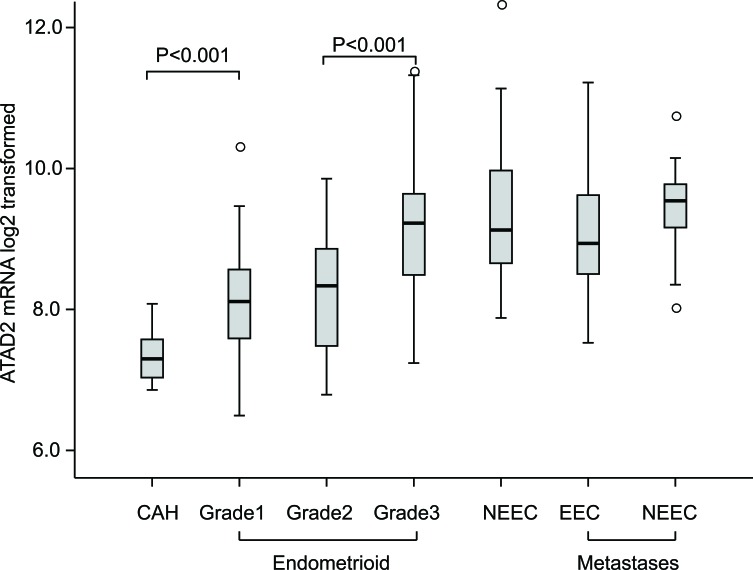
*ATAD2* mRNA expression is elevated in aggressive stages of endometrial cancer A significant increase in *ATAD2* expression was found from complex atypical hyperplasias (CAH; *n* = 18) to grade 1 (*n* = 49) endometrioid endometrial carcinomas (EEC) (*P* < 0.001), with a further increase from grade 2 (*n* = 53) to grade 3 (*n* = 39) EECs (*P* < 0.001). Non-endometrioid (NEEC, *n* = 34) tumors showed the same high levels of *ATAD2* as grade 3 EEC. *ATAD2* expression is high in metastases from both EECs (*n* = 22) and NEECs (*n* = 19).

High ATAD2 expression has been linked to aggressive disease in breast cancer and endometrial cancer but little is known regarding the variation in *ATAD2* mRNA expression related to different stages of disease development for endometrial cancer. We investigated the mRNA levels of *ATAD2* in a unique collection of fresh tissue from 18 precursor cancer lesions (complex atypical hyperplasias, CAHs), 141 primary endometrial cancer lesions of the endometrioid subtype and 34 of non-endometrioid subtypes, as well as 42 metastatic lesions (Figure 1). We find that the expression of *ATAD2* increases in early stages of cancer progression, with a significant increase in *ATAD2* expression from CAH lesions to grade 1 endometrioid lesions (*P* < 0.001) and a further significant increase from grade 1/2 to grade 3 endometrioid endometrial cancers (*P* < 0.001). *ATAD2* mRNA levels are equally high in grade 3 endometrioid lesions, non-endometrioid and metastatic lesions. We have previously shown by SNP analyses of 70 cases that the increase in *ATAD2* associates with amplification of the 8q24 region [16]. To further explore possible underlying mechanisms for increased ATAD2 levels we utilized publicly available data from the The Cancer Genome Atlas (TCGA) consortium to investigate specific *ATAD2* copy number alterations and DNA methylation status of *ATAD2*. In the pan cancer dataset, accessed through the TCGA copy number portal, *ATAD2* was frequently amplified in 8 out of 11 total cancer types (including endometrial cancer, Supplementary Table S1), with the overall frequency of amplification of 31%. However, these amplifications were not due to the *ATAD2* gene specifically being amplified, but rather larger regions within the 8q24 region containing the gene. The event of deletions (frequency of 2%) was not significant in any cancers across the pan cancer dataset. To explore *ATAD2* alterations in detail for endometrial cancer, putative copy-number alterations (GISTIC-based analysis [17]), showed a highly significant correlation of increased copy-number levels and mRNA expression of *ATAD2* for endometrial cancer, confirming previous findings that *ATAD2* amplification leads to higher expression of ATAD2 (Supplementary Figure S1A). In contrast, when investigating the DNA methylation status of *ATAD2* (through 13 specific *ATAD2* probes available in the k450 DNA methylation dataset from TCGA), we found the gene to be almost completely hypomethylated, suggesting that loss of DNA methylation is not a plausible mechanism providing elevated ATAD2 levels in endometrial cancer (Supplementary Figure S1B).

### Immunohistological staining of ATAD2 is associated with reduced disease-specific survival, and correlates well with mRNA expression levels

**Figure 2:**
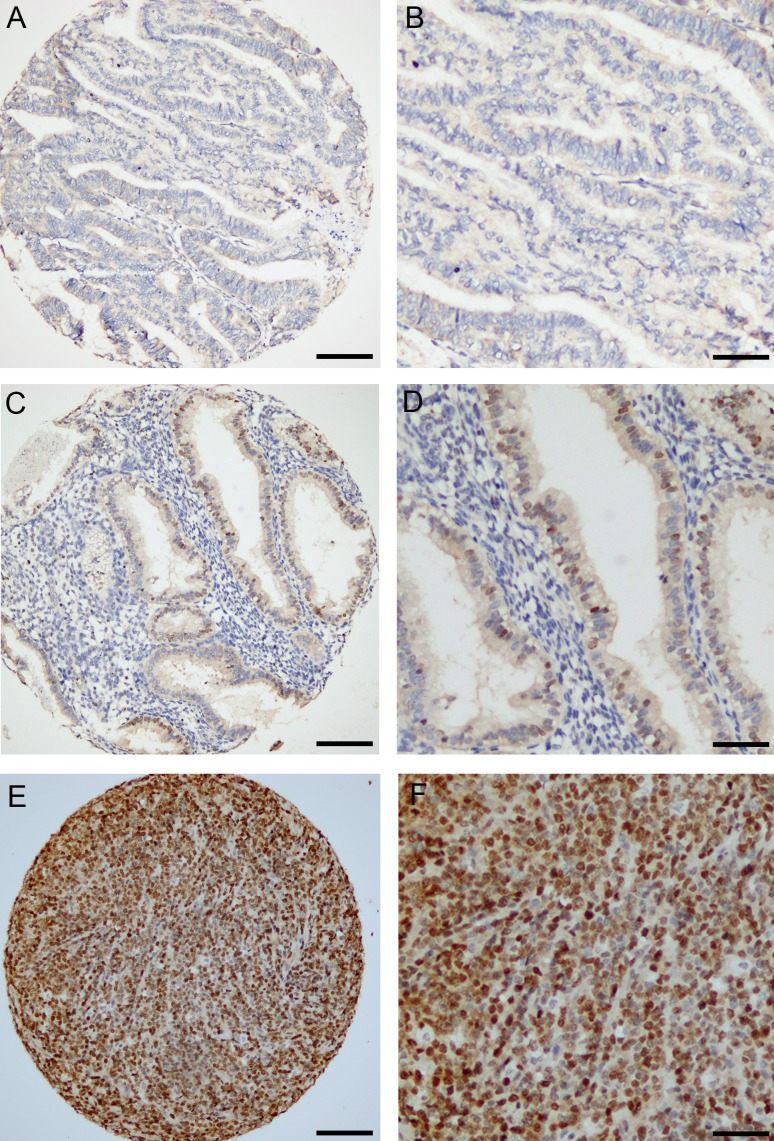
Immunohistochemical detection of ATAD2 protein in endometrial carcinomas ATAD2 was mainly nuclear, with low expression detected in 50% of all cases (**A.**; **B.** high magnification). 32% of the cases showed intermediate expression (**C.**; **D.** high magnification) and 18 % had high expression of ATAD2 (**E.**; **F.** high magnification). Details regarding IHC staining and scoring are given is the methods. Bar = 100&micro;m for A, C and E, Bar = 50&micro;m for B, D and F.

**Figure 3:**
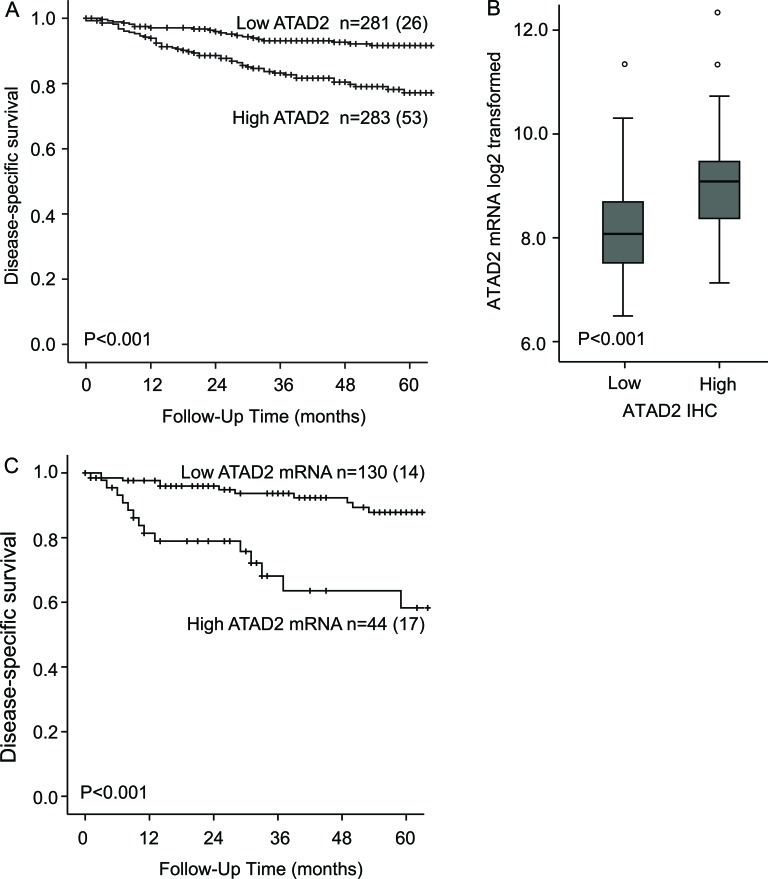
ATAD2 predicts poor prognosis in endometrial cancer High expression of ATAD2 protein significantly predicts poor prognosis in endometrial cancer compared to low ATAD2 expression **A.**. Patients with high ATAD2 protein levels (*n* = 76) have significantly higher *ATAD2* mRNA expression than patients with low ATAD2 protein expression (*n* = 79) **B.**. Poor outcome is also predicted by high ATAD2 mRNA levels, defined as the upper quartile **C.** Number in brackets in figure A and C represents number of disease specific deaths in each group.

To investigate the prognostic impact of ATAD2 protein level in endometrial cancer, 564 primary tumors were stained by immunohistochemistry. ATAD2 is detected in the nuclei of cancer cells, with little staining in cytoplasm or surrounding stromal tissue (Figure 2). Only nuclear staining was recorded. 50% of all cases showed low expression (Index 0, 1 and 2; Figure 2A and 2B), 32% showed intermediate expression (Index 3 and 4; Figure 2C and 2D) and 18% showed high expression (Index 6 and 9; Figure 2E and 2F) of ATAD2. For subsequent analyses, intermediate and high expression groups were combined according to similar survival pattern. Details regarding scoring of ATAD2 are given in the method section. When comparing ATAD2 expression with clinical and histopathological variables for aggressive endometrial cancer, we find a highly significant association between high ATAD2 expression and high FIGO (International Federation of Gynecology and Obstetrics) stage, non-endometrioid subtype, high grade and aneuploidy (Table 1; all *P*-values ≤0.001). ATAD2 expression is also negatively correlated with protein expression of hormone receptors ERα (*P* < 0.001), PR (*P* < 0.001) and Androgen Receptor (AR; *P* = 0.009), indicating that ATAD2 may be a biomarker relevant to identify patients unlikely to respond to antihormonal treatment. In survival analysis (Figure 3A), high ATAD2 significantly predicts poor disease-specific survival in univariate analysis (*P* < 0.001).

**Table 1:** ATAD2 protein expression in prospectively collected tumors related to clinico-pathologic factors in 564 patients with endometrial carcinoma

	ATAD2^a^		
Variable	Low n (%)	High n (%)	P-value
Age (mean)			0.108
<66	156 (53)	138 (47)	
≥66	125 (46)	145 (54)	
FIGO-09 stage			0.001
I-II	251 (53)	223 (47)	
III-IV	30 (33)	60 (67)	
Histologic type			<0.001
Endometrioid	253 (55)	210 (45)	
Serous	9 (21)	34 (79)	
Clear cell	12 (57)	9 (42)	
Carcinosarcoma	5 (19)	22 (81)	
Undifferentiated/Others	2 (20)	8 (80)	
Histologic grade*			<0.001
Grade 1	133 (66)	70 (34)	
Grade 2	92 (53)	81 (47)	
Grade 3	25 (30)	58 (70)	
Metastatic nodes			0.08
Negative	205 (50)	207 (50)	
Positive	20 (37)	34 (63)	
Ploidy			0.001
Diploid	158 (53)	142 (47)	
Aneuploid	26 (32)	56 (68)	
P53			<0.001
Low	171 (60)	116 (40)	
High	30 (31)	66 (69)	
PR			<0.001
Positive	245 (58)	177 (42)	
Negative	34 (25)	104 (75)	
ERα			<0.001
Positive	231 (55)	192 (45)	
Negative	45 (33)	90 (67)	
AR			0.009
Positive	171 (53)	152 (47)	
Negative	70 (41)	102 (59)	

a Low=index 0-2, high=index 3-9

* Endometrioid Endometrial cancers only

The validity and robustness of antibodies to detect prognostic biomarkers is debated due to the possibility for unspecific staining. Expression data from 155 overlapping patients is available from microarrays of the 564 patients explored for protein level by immunohistochemistry. We find a highly significant correlation between mRNA and protein levels (*P* < 0.001, Figure 3B) supporting that immunohistochemical staining adequately reflects mRNA level. In line with this, grouping mRNA in accordance with IHC median cutoff into two equal patient groups is significantly associated with poor prognosis, with a 5-years disease specific survival of 72% compared to 88% for patients with high ATAD2 mRNA (*p* = 0.006). For mRNA, applying the upper quartile limit demonstrates to have an even stronger prognostic impact with a 5-years disease specific survival of 58% compared to 88% for patients with high ATAD2 mRNA (*P* < 0.001, Figure 3C) suggesting a potential for using mRNA assessment as an even stronger prognostic marker, although currently less applicable in a routine clinical setting. This is in line with our previous observation in a smaller dataset [16]. Taken together, our findings support that immunohistochemical staining for ATAD2 represent a clinically applicable method to measure phenotype relevant ATAD2 level in endometrial carcinomas, also with the advantage of morphologic assessment during the IHC analysis.

### ATAD2 is associated with poor prognosis in the subgroup of ER positive endometrial cancers, and is an independent marker for poor prognosis in endometrioid endometrial cancer patients

**Figure 4:**
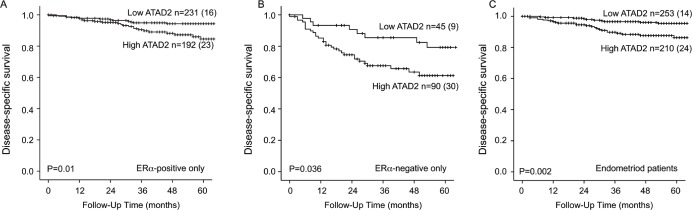
ATAD2 is a prognostic marker in subgroups of endometrial cancer High expression of ATAD2 identifies patients with poorer survival within the ERα positive subgroup **A.**, the ERα-negative subgroup **B.** and also in the endometrioid only subgroup **C.**. Number in brackets represents number of disease specific deaths in each group.

We investigated the potential of ATAD2 as a biomarker for poor prognosis within subgroups of endometrial cancer. We found ATAD2 to significantly predict poor survival in both ERα positive (Figure 4A) and ERα negative (Figure 4B) patients. ATAD2 has been suggested as a cofactor for ERα and therefore we focused specifically on the prognostic impact of ATAD2 in the ERα positive subgroup of patients. Interestingly, we find that high ATAD2 is significantly associated with reduced disease-specific survival (Figure 4A) and correlates with non-endometrioid type, high grade, aneuploidy and loss of PR receptor (all P-values≤0.002; Table 2) also in the subgroup of ERα positive patients.

**Table 2:** ATAD2 protein expression in prospectively collected tumors related to clinico-pathologic factors in 423 Estrogen Receptor α positive patients with endometrial carcinoma

	ATAD2^a^		
Variable	Low n (%)	High n (%)	P-value
Age (mean)			0.690
<66	126 (56)	101 (44)	
≥66	105 (54)	91 (46)	
FIGO-09 stage			0.15
I-II	211 (56)	167 (46)	
III-IV	20 (44)	25 (56)	
Histologic type			0.001
Endometrioid	220 (57)	166 (43)	
Non-endometrioid	11 (30)	26 (70)	
Histologic grade			<0.001
Grade 1/2	198 (60)	131 (40)	
Grade 3	30 (33)	60 (67)	
Metastatic nodes			0.798
Negative	172 (53)	155 (47)	
Positive	13 (50)	13 (50)	
Ploidy			0.002
Diploid	137 (57)	104 (43)	
Aneuploid	15 (32)	32 (68)	
PR			<0.001
Positive	219 (59)	155 (41)	
Negative	11 (23)	36 (77)	

a Low=index 0-2, high=index 3-9

ATAD2 also proved to be significantly associated with poor survival for patients with endometrioid histology (Figure 4C). Cox analysis was performed for the endometrioid subgroup since an interaction between FIGO stage and histologic type was suspected. We find that ATAD2 independently predicts poor survival in the endometrioid subgroup, in a Cox regression model adjusting for age, FIGO stage and grade (Table 3).

**Table 3:** Multivariable survival analyses of endometrioid endometrial cancer patients according to Cox’ proportional hazards regression model

Variable	n*	Unadj. HR	95% CI	P-value	Adj. HR	95% CI	P-value
**Age (mean=63)**				<0.001			<0.001
	459	1.06	1.02-1.09		1.03	1.02-1.09	
**FIGO stage**				<0.001			<0.001
I/II	407	1	-		1	-	
III/IV	52	19.4	9.8-37.9		17.1	8.4-34.8	
**Histological grade**				0.001			0.67
Grade 1-2	376	1	-		1	-	
Grade 3	83	3.13	1.6-6.1		1.17	0.56-2.42	
**ATAD2**				0.003			0.02
Low expression	250	1	-		1	-	
High expression	209	2.87	1.44-5.69		2.39	1.15-4.99	

* Cases with data available for all variables included in uni- and multivariable analyses

### Primary tumors with high ATAD2 demonstrate changes in cell cycle regulation and enrichment for B-MYB-associated pathways

When examining the most significantly upregulated genes in primary tumors with high ATAD2 protein levels (false discovery rate; FDR < 0.05 in SAM analysis), several key regulators of cell proliferation are amongst the top 100 regulated genes (Supplementary Table S2). Amongst these are genes directly linked to mitosis, cell division and cell signaling. For endometrial carcinoma patients with high ATAD2 levels, 9 out of 20 genes known to be directly regulated by ATAD2 in breast cancer [7], are found to be highly expressed (highlighted in red and listed in Supplementary Table S2). We further explored the Connectivity Map database using this genelist to search for drugs with treatment potential for the patient group with high ATAD2 protein level, identifying HDAC and PI3K-pathway inhibitors amongst the to ranked drugs (Supplementary Table S3). To further explore the molecular signaling pathways altered in tumors with high ATAD2 protein levels, GSEA analysis was performed. Tumors with high ATAD2 protein levels show signs of increased proliferation, with gene sets linked to cell cycle in the C5 MSigDB collection (Gene Ontology gene sets) highly enriched (Table 4, top panel). To gain more insight in the molecular processes underlying this, we also explored the C2 MSigDB, representing curated gene sets generated from online pathway databases and PubMed publications. Among the four gene sets significantly enriched (FDR < 0.05) in tumors with high ATAD2, three gene sets pointed to B-MYB; the two B-MYB gene sets published by Shepard *et al* [18], as well as the TFRC targets dn published by O’Donnell *et al* [19]. In addition one gene set linked to prostaglandin and E2 response was significantly enriched in patients with high ATAD2. To validate the link between high ATAD2 and B-MYB signaling pathways, we explored a publically available external dataset confirming similar enrichment for two B-MYB gene sets and the TFRC targets dn all being amongst the top 5 ranked gene sets related to high ATAD2 level (Supplementary Table S4).

**Table 4:** Gene sets significantly enriched in patients with high ATAD2 protein expression in gene set enrichment analysis (GSEA)

Rank	Gene set name-MSigDB C5	*n*	NES	FDR*
1	Mitotic Cell Cycle	139	2.12	0.022
2	Cell Cycle Process	174	2.08	0.025
3	Cell Cycle Phase	155	2.05	0.024
4	Interphase	64	2.04	0.023
5	M-phase of mitotic cell cycle	78	2.0	0.034
6	Interphase of mitotic cell cycle	58	2.0	0.028
7	M-phase	102	2.0	0.025
8	S-phase	12	1.99	0.030
9	Mitosis	76	1.98	0.029
10	Cell cycle	288	1.95	0.041
11	DNA dependent DNA replication	47	1.95	0.040
12	Cell cycle checkpoint	44	1.94	0.041

* Gene sets with FDR<0.05 included

**Table:** 

Rank	Gene set name- MSigDB C2	*n*	NES	FDR
1	Shepard BMYB targets [18]	66	2.21	0.026
2	Shepard BMYB morpholino DN [18]	181	2.19	0.016
3	Chemnitz response to prostaglandin E2 UP [35]	124	2.18	0.015
4	ODonnell TFRC targets DN [19]	118	2.12	0.038
5	Dutertre estradiol response 24HR UP [36]	294	2.09	0.057
6	Kaufmann DNA replication genes [37]	132	2.08	0.054

**Figure 5:**
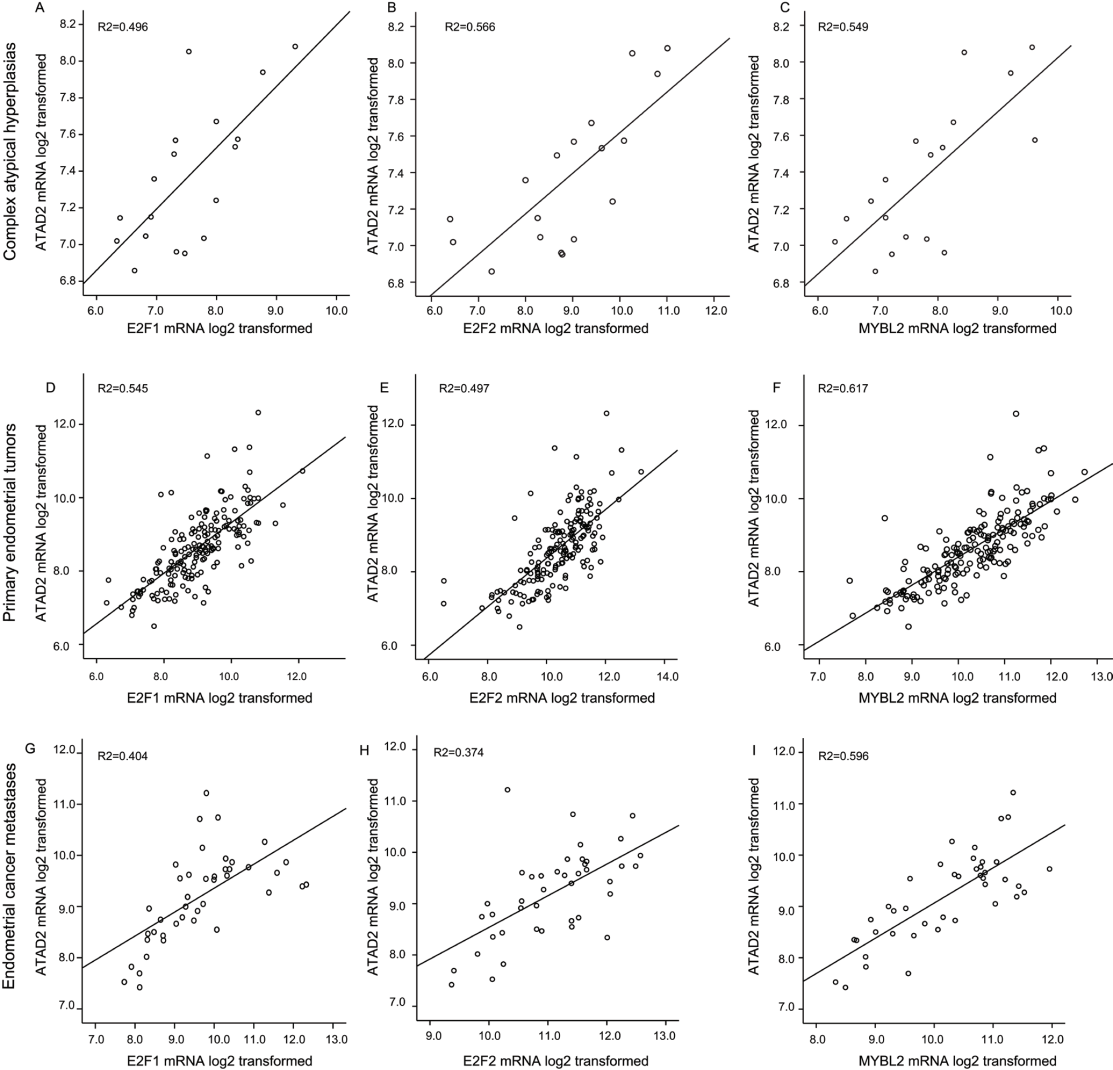
Expression of *ATAD2* strongly correlates with E2Fs and MYBL2 levels The mRNA levels of the transcription factors E2F1 and E2F2 as well as their downstream target MYBL2 (protein name B-MYB) is strongly correlated with expression levels of *ATAD2*. These patterns for strong correlations are consistently seen during tumor development from precursor lesions of endometrial cancer, complex atypical hyperplasias (**A.**-**C.**; *n* = 18), through primary tumors (**D.**-**F.**; *n* = 176) to metastatic lesions (**G.**-**I.**; *n* = 42).

It has been proposed that elevation of ATAD2 may lead to a loop of transcriptional overexpression of its downstream targets such as E2Fs and ATAD2 itself [13]. ATAD2 is both a target for, and interacts with E2F, regulating its activity. The E2Fs transcription factors direct transcription of B-MYB (gene name *MYBL*) during cell cycle progression [20]. To explore this reported relation in different stages of endometrial cancer, we examined the mRNA levels separately in CAHs, primary tumors and metastatic lesions. We find a consistent and highly significant correlation between the gene levels for *ATAD2* and *E2F1*, *E2F2* and *MYBL* during all stages of disease from precursors to metastatic lesions (Figure 5). *ATAD2* amplification is most often seen in the Copy number high (serous like) subtype of the TCGA molecular subclasses (Supplementary Figure S2 A and B). This same pattern is also observed for *E2F1* and *MYBL2*, located close to the region 20q13 often amplified in the serous like molecular sub group of endometrial cancer [11], indicating that in addition to a transcriptional link, these genes may also be co-amplified due to general genomic instability.

## DISCUSSION

Recent studies suggest that *ATAD2* is overexpressed in several cancers and that *ATAD2* may have a potential both as a biomarker and as a therapeutic target. We have previously identified and also recently validated *ATAD2* as one of 29 genes predicting recurrence in an endometrial cancer recurrence score [11, 21]. Also *ATAD2* mRNA level has been linked to MYC expression and amplification of the 8q24 region [16]. Here, we further explore expression of *ATAD2* mRNA and the protein levels of ATAD2 by IHC and investigate its potential as a prognostic marker in endometrial cancer. We find that *ATAD2* is low in CAH lesions, in line with the apparent no expression in normal uterine tissue [5]. Compared to the precursor lesions CAH, we see a significantly increase in *ATAD2* in grade 1 endometrioid cancers with a further increase to grade 3 endometrioid endometrial cancers. *ATAD2* levels are high also in non-endometrioid endometrial cancers and metastatic lesions. The mechanism involved in the observed increase in ATAD2 level during cancer development is only partially understood. Elevated levels of *ATAD2* have been linked to gene amplification. Although DNA methylation could represent an additional mechanism contributing to increased expression, we do not find sufficient evidence that this is the case investigating publically available TCGA data. This is contrasting the significant association between the increase in mRNA expression and increase in gene copy number, supporting that gene copy number changes are the most dominant mechanism causing the elevated expression of ATAD2 observed in endometrial cancers.

We have applied the previously validated antibody to detect ATAD2 protein expression in endometrial cancer in relation to clinico-pathological parameters. We find that high expression of ATAD2 predicts poor outcome and significantly associates with markers for aggressive endometrial cancer, such as high FIGO stage and high grade, non-endometrioid type, and loss of hormone receptors AR, PR and ERα. This is in line with findings from breast cancer, where high ATAD2 detected by IHC is associated with aggressive disease [7]. We find ATAD2 to be an independent prognostic marker in endometrioid tumors and high ATAD2 corresponds with aggressive disease for ERα positive patients. *ATAD2* has been described as an estrogen and androgen responsive gene in hormone dependent cancer cells and a co-activator for full activity of ER and AR [4, 14], contributing to cancer related proliferation. Our results may point to a similar regulation of ATAD2 also in endometrial cancer. However, additional mechanistic impact by ATAD2 is evident given its high expression detected also in the often ER negative non-endometrioid tumors.

Molecular profiling of patients according to ATAD2 level was performed to explore genetic alterations associated with increase in ATAD2 expression. In concordance with findings from breast cancer [7] we find that ATAD2 overexpression correlate strongly with expression of genes associated with increased proliferation, suggesting that ATAD2 either act as an upstream mediator of their expression or as a collaborator of their function. GSEA analyses using the GO gene sets (MSigDB C5) showed enrichment of gene sets linked to cell proliferation in patients with high ATAD2 levels (Table 4). A more specific GSEA analysis using curated data sets (MSigDB C2) identified gene sets linked to B-MYB signatures [18] as well as TFRC (Transferrin receptor) and estradiol treatment as enriched in patients with high ATAD2 in our patient cohort. This was validated in an external dataset. In SAM analyses of primary tumors we also found B-MYB (listed as MYBL2), E2F1 and E2F2 to be amongst the top 20 up regulated genes in primary tumors with high ATAD2. This strong association of expression of E2F1, E2F2 and MYBL2 with ATAD2 is found in both CAHs, primary and metastatic endometrial cancer lesions, indicating that the expression of these genes are tightly linked in all stages of endometrial cancer. This is interesting, since links between ATAD2, B-MYB and E2Fs has been reported also in other cancers. In breast cancer, E2F1 protein regulates ATAD2 gene expression [6] and B-MYB has been reported to have a role in ATAD2 driven cell proliferation [7]. B-MYB belongs to the MYB family of transcription factors and is involved in regulation of cell cycle at several levels [22]. B-MYB is also shown to cooperate with E2F transcription factors to regulate genes important for G_2_/M transition [20]. Also in prostate cancer E2F1 binds the ATAD2 gene regulatory region to activate its gene transcription [23]. A similar relationship between E2Fs, ATAD2 and B-MYB has not been investigated in endometrial cancer. However our data support that these genes cooperate to drive proliferation, resulting in aggressive disease. As already shown in breast cancer, we also find several members of the Kinesin protein family (KIF11, KIF15, KIF23, KIF2C and KIFC1) to be upregulated in patients with high expression of ATAD2. Kinesins have important roles in cell division and signaling, and ATAD2 has been shown to regulate expression of key kinesin members, both in the presence and absence of estrogen in breast cancer cell lines [24]. Aberrant expression of kinesins has also been linked to resistance to taxol treatment and attempts have been made to target these proteins in cancer [25]. Given the high number of kinesins with altered expression in our study, a more detailed study should be undertaken to pinpoint the specific role of the different family members in endometrial cancer.

Several prognostic gene signatures defined in cancer include *ATAD2*. Some have been prospectively validated to reflect poor outcome [21]. In addition, we and others have shown that increased *ATAD2* may predict survival, and we here confirm that ATAD2 protein assessed by IHC is an independent prognostic marker in endometrioid endometrial cancer patients. Combined with the increased efforts to target bromodomain-containing proteins in cancer [15, 26], ATAD2 seems an attractive cancer protein for more detailed studies. It is also interesting that our molecular analyses point to PI3K-pathway and HDAC inhibitors as potential drugs for treatment of patients with high ATAD2. This is in line with previous observations regarding treatment of aggressive molecular subclasses of endometrial cancers, but needs further validation in a clinical setting, possibly alongside validation of ATAD2 as a biomarker for endometrial cancer.

The molecular mechanisms underlying the aggressive behavior of tumors with high ATAD2 is still poorly understood in endometrial cancer. However our data points to changes in regulators of cell cycle progression as potential key players. Identifying the relationship between these as well as the other major downstream effectors may point to development and/or testing of novel therapeutics in endometrial carcinoma clinical trials.

## MATERIALS AND METHODS

### Ethics statement

All parts of the study have been approved according to Norwegian legislation as well as international demands for ethical review. Patients were included in the study after written informed consent approved by the ethics committee (REK West). The study was approved by the Norwegian Data Inspectorate, Norwegian Social Sciences Data Services, and the Western Regional Committee for Medical and Health Research Ethics, REC West (NSD15501; REK 052.01).

### Tumor specimens

A patient series counting 585 patients diagnosed with primary endometrial carcinoma in Hordaland County (Norway) during the period 2001-2012 were prospectively collected. Patients were staged according to FIGO 2009 criteria and age at diagnosis, histologic type, histologic grade, treatment and follow-up were obtained from the clinical records and routine histopathology reports from a tumor board setting. Formalin-fixed paraffin-embedded (FFPE) tissue from 564 patients was used for immunohistochemistry, while fresh frozen tissues from 18 CAH, 176 primary endometrial cancers (155 overlapping with TMA cases) and 42 metastatic lesions were available for mRNA extraction. The metastatic lesions were from 31 individual patients, where 26 patients had available tissue from both the primary lesion and the metastases in parallel. 6 metastatic lesions from 5 individual patients missing a primary tumor counterpart, were also included in the analyses. Detailed information on the metastatic samples is included in Supplementary Table S5.

### External datasets

*ATAD2* gene copy number alterations and *ATAD2* methylation status in relation to mRNA levels were investigated using the publicly available endometrial carcinoma data from The Cancer Genome Atlas (TCGA). Data was downloaded through the Cancer Browser (https://genome-cancer.ucsc.edu. Dataset details: DNA methylation (hMethyl450_2014-05-02), RNA expression (exp_GAV2_2014-08-28) and CNA (gistic2thd_2014-08-22).

For external validation of findings in microarray analyses, a dataset from 111 tumors was obtained from the Expression Project for Oncology (expO: http://www.intgen.org). Gene Expression Omnibus (GEO) accession numbers and information on clinico-pathologic data as well as processing of this dataset to construct transcript level probe sets have previously been described [11, 27].

### Immunohistochemistry

FFPE tissue from 564 patients was used to construct tissue microarrays (TMAs) as previously described [28]. Briefly, three cylinders of 0.6 mm were retrieved from high tumor purity areas using a custom-made precision instrument (Beecher Instruments, Silver Spring, MD, USA) and mounted in a paraffin block. TMA sections (5&micro;m) were stained for ATAD2 expression using a validated antibody and a protocol described previously [7]. AR expression was detected using ab133273 (Abcam) diluted 1:100 for 1 hour at room temperature followed by 30 min incubation with secondary HRP-conjugated antibody. Diaminobenzidine was applied for 8 min, before counterstained with hematoxylin. The immunostained sections were scored visually by light microscopy, blinded for the patients clinical characteristics and outcome, using a semi quantitative and subjective method described in more detail previously [29]. Tumor tissue in all three cylinders were assessed to estimate the average protein staining. Briefly, a staining index was calculated as a product of staining intensity (0-3) and area of positive tumor cells (1≤10%, 2 = 10-50% and 3≥50%). In subsequent statistical analyses, indexes were grouped according to similarity in survival and considering the size of the subgroups and the number of events in each category. Index 0-2 (*n* = 281) was considered low, index 3-9 (*n* = 283) was considered high. Hormone receptor status was defined by IHC and recorded using the same soring system. Index 0-2 was defined as ERα negative status while index 3-9 as ERα positive. For PR and AR status, index 0 was considered negative while index 1-9 positive. Details related to scoring methods applied and approaches to cut point development for ER and PR hormone receptor status have been reported previously [30, 31]. P53 protein expression status was available for 383 patients with ATAD2 protein status. P53 IHC protocol and scoring has been published previously [32].

### Gene expression analyses

Gene expression alterations in relation to ATAD2 expression were investigated in fresh tissues from 18 CAH, 176 primary endometrial cancers and 42 metastatic endometrial cancer lesions derived from 31 patients. Expression data from a subset of these patients from 122 of the primary tumors and 19 metastases have previously been explored in relation to copy number changes for the MYC region in endometrial cancer [16]. Samples were validation for high tumor cell content [33]. Areas with necrosis were avoided. RNA was extracted using the RNeasy Mini Kit (Qiagen, Hilden, Germany) and hybridised to Agilent Whole Human Genome Microarrays 44k (Cat.no. G4112F), according to the manufacturer’s instructions. Microarrays were scanned using the Agilent Microarray Scanner Bundle. Normalization of raw data and expression analyses were performed using J-Express software (Molmine, Bergen, Norway). Expression data were quantile normalized. Median spot signal was used as intensity measure. For survival analyses using mRNA values, values were grouped either according to IHC cutoff (two equally sized patient groups) or in quartiles, where the upper quartile was defined as high expression. Differentially expressed genes in tumors with high levels of ATAD2, detected by IHC, were identified using the Significance Analysis of Microarray (SAM) method. 275 genes were identified with False discovery rate (FDR) value < 0.05. Two class unpaired gene set enrichment analysis (GSEA) was performed using SAM score as scoring method and balanced sample permutations. The Molecular signature database v4.0 (MSigDB) C2 Curated gene set collection and C5 GO gene sets were used (http://www.broadinstitute.org/gsea/msigdb/index.jsp). Only gene sets with FDR < 0.05 was considered significantly enriched. To validate the findings from our dataset, an external dataset with mRNA values from 111 endometrial cancer patients was investigated to compare transcriptional alterations related to high versus low ATAD2 level. Given the high correlation between ATAD2 protein measured with IHC and mRNA levels found in our dataset, median level of ATAD2 was defined as cut point in the external dataset in accordance with detected IHC cutoff. The correlation between global gene expression pattern and potential new therapeutics for tumors with high ATAD2 protein was assessed using the drug signatures database Connectivity Map [34] as previously described [27]. The microarray data is publicly available at ArrayExpress, with accession number E-MTAB-2532.

### Statistical analysis

Statistical analyses were done using IBM SPSS Statistics software version 21 (IBM, Armonk, NY, USA). All tests were two sided, and probability of < 0.05 was considered statistically significant. For categorical variables groups were compared using Pearson chi-square test, and Mann-Whitney U test was used to test correlations for continuous variables. Univariate survival analyses of time to death due to endometrial carcinoma (disease specific survival) were performed using the Kaplan-Meier (product-limit) method. Entry date was defined as date of primary surgery, and patients who died from other causes were censored at the date of death. Differences in survival between groups were estimated by the log-rank (Mantel Cox) test. The prognostic impact of ATAD2 adjusted for other prognostic markers was evaluated by the Cox proportional hazard regression models. All variables included were examined by log minus log plots to assure that the assumptions of proportional hazards were satisfied. Since an interaction between histologic type and FIGO stage was suspected, the Cox analysis was performed for the endometrioid subgroup.

## SUPPLEMENTARY MATERIAL FIGURES AND TABLES


