# Supplementary material for: ATAD2 overexpression links to enrichment of B-MYB-translational signatures and development of aggressive endometrial carcinoma
Source: Oncotarget. 2015 Jul 22;6(29):28440–52. doi: 10.18632/oncotarget.4955 (PMC4695070; doi:10.18632/oncotarget.4955)
Supplement: Supplementary file 1 [file oncotarget-06-28440-s001.pdf]

ATAD2 overexpression links to enrichment of B-MYB-translational signatures and development of aggressive endometrial carcinoma

Supplementary Material

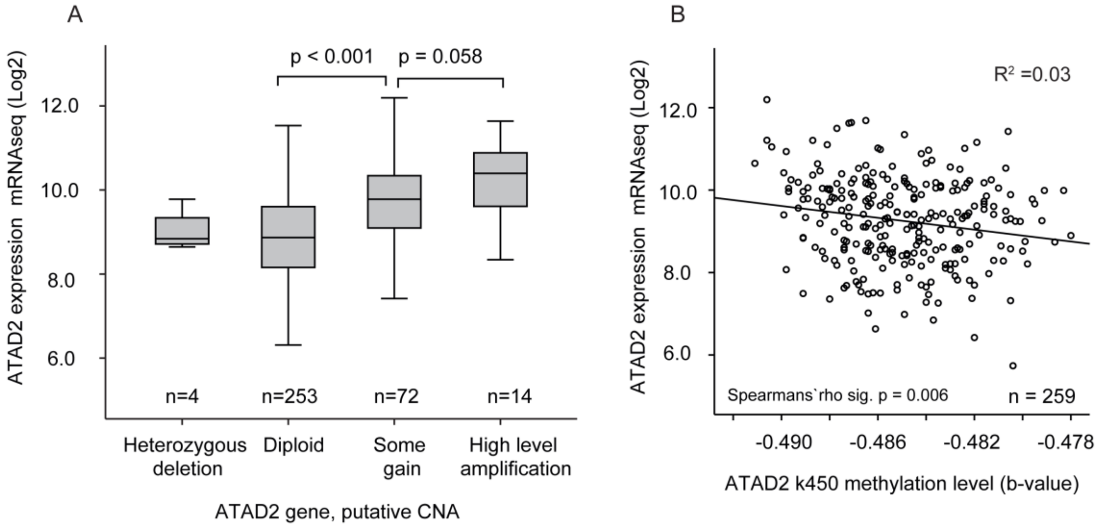

Supplementary Figure S1:

Level of mRNA expression of *ATAD2* significantly correlates with *ATAD2* gene copy-number alterations and is not dependent on change in methylation status.

The TCGA data from endometrial carcinomas (http://www.broadinstitute.org/tcga/home) was enquired for a possible link between mRNA expression levels and copy number alterations (CNA). There is a significant increase in *ATAD2* mRNA expression upon increase in copy-numbers (some gain, high level amplification) in endometrial cancer (A). ATAD2 gene shows low methylation level. Out of 13 total probes, 3 probes are inversely correlated with *ATAD2* mRNA expression. Data range from +0.5 to -0.5, presenting high level of methylation to low level of DNA methylation, respectively. Figure presents data from the most significantly inversely correlated probe (cg17093362, Chr 8: 124477902) (B).

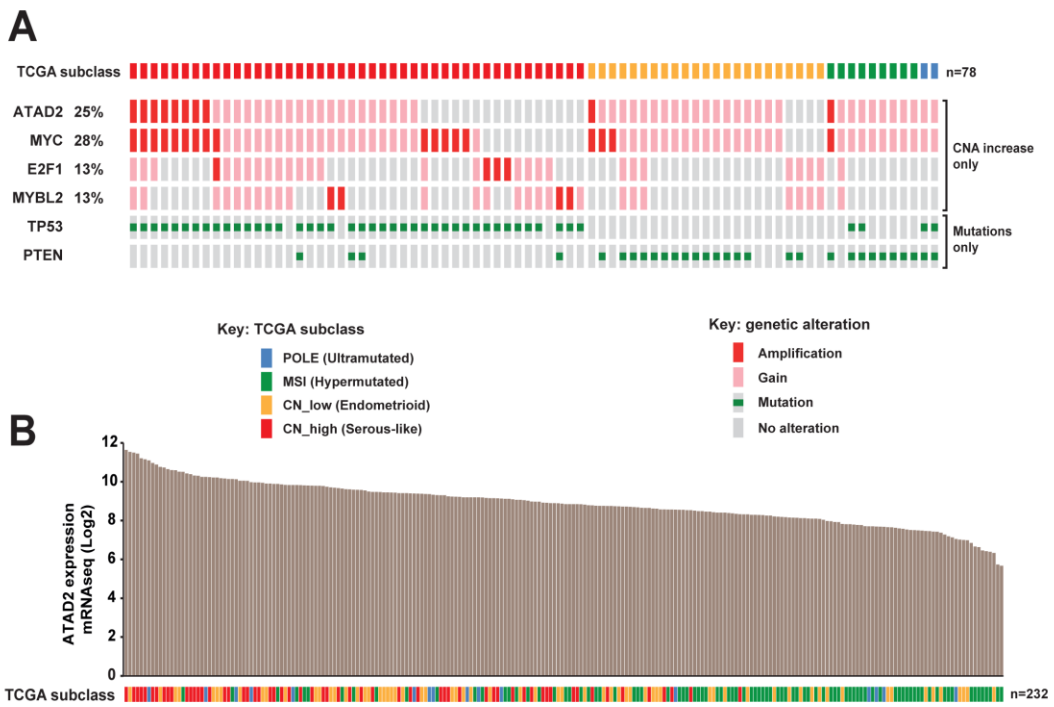

Supplementary Figure 2.

ATAD2 copy number distribution and RNA expression towards TCGA subclassification. Distribution of copy number increase of ATAD2 in relation to that of MYC, E2F1 and MYBL2. ATAD2 copy number increase occurs more frequently in the copy number high (serous-like) and copy number low (Endometrioid) subgroups, affecting 47% and 21% cases within these subgroups, compared to 14% in the MSI subgroup and POLE 12% of the POLE subgroup. Note high degree of coamplification between ATAD2 and MYC, and E2F1 and MYBL2, again also most frequently amplified in the copy number high/low subgroups. Mutation status is shown for TP53 anf PTEN for reference purposes. Percent affected cases in total are indicated next to gene name. Only cases with alteration are shown in figure (n=78/232). The figure is generated through http://www.cbioportal.org, with modifications (A).

Rank order of ATAD2 RNA seq expression data for all cases with TCGA subclass annotation (n=232). The cases with the highest ATAD2 expression falls into the copy number high (serous-like) subclass of TCGA, in agreement with these cases often showing ATAD2 copy number increase (B)

Supplementary Table S1:

Amplification of ATAD2 (chr8:124332090-12448705) by pan cancer GISTIC copy number data[a]

Cancer subset	In peak?	Position of nearest peak on chr8 [b]	#Genes in peak	Q-value[c]	Frequency of amplification			#Cases
					Overall	Focal	High-level	
**All cancers combined**	No	128740968 - 128758521	1	1.1E-108	0.4392	0.1121	0.0521	4934
**Kidney clear cell carcinoma**	Yes	118410626 - 145232496	202	0.438	0.1167	0.0161	0	497
**Ovarian serous carcinoma**	No	128696277 - 129683434	7	3.96E-81	0.6892	0.3854	0.1474	563
**Breast cancer**	No	128668369 - 128777531	1	1.37E-17	0.5975	0.117	0.1319	872
**Colorectal cancer**	No	128507651 - 128533931	0	6.2E-09	0.5402	0.0718	0.041	585
**Lung squamous cell carcinoma**	No	128343452 - 128420241	0	9.58E-07	0.5727	0.1541	0.032	344
**Uterine and endometrial carcinoma**	No	128740968 - 128781251	1	1.74E-06	0.3145	0.0766	0.0141	496
**Head and neck squamous cell carcinoma**	No	128283618 - 128408116	0	2.12E-05	0.6774	0.1161	0.0226	310
**Lung adenocarcinoma**	No	129166547 - 129190290	0	3.22E-05	0.5322	0.0868	0.0224	357
**Bladder cancer**	No	127581610 - 129572525	10	0.0926	0.4559	0.1103	0.0074	136
**Acute myeloid leukemia**	No	No peak on chr.	0	0.938	0.1134	0.0052	0	194
**Glioblastoma multiforme**	No	128025053 - 130346811	11	1	0.081	0.0172	0.0017	580

[a]Assessed through the TCGA copy number portal, by analysis version 2013-08-16 Pan cancer data set. Data concerning ATAD2 deletions are not shown. Additional info on interpretation available at website: http://www.broadinstitute.org/tcga/home). [b]Nearest peak located 4.33 Mb away. [c]ATAD2 is significantly focally amplified ($Q \leq 0.25$) in 8 out of 11 cancers (red).

Supplementary Table S2:

Gene Name	Systematic Name	Description	Fold Change	FDR
AA019203	AA019203	ze57f04.s1 Soares retina N2b4HR Homo sapiens cDNA clone IMAGE:363103 3' similar to gb:X62534 HIGH MOBILITY GROUP PROTEIN HMG2 (HUMAN);, mRNA sequence [AA019203]	1.834	<0.001
E2F1	NM_005225	Homo sapiens E2F transcription factor 1 (E2F1), mRNA [NM_005225]	1.803	<0.001
RECQL4	NM_004260	Homo sapiens RecQ protein-like 4 (RECQL4), mRNA [NM_004260]	1.787	<0.001
TYMS	NM_001071	Homo sapiens thymidylate synthetase (TYMS), mRNA [NM_001071]	1.778	<0.001
C15orf42	NM_152259	Homo sapiens chromosome 15 open reading frame 42 (C15orf42), mRNA [NM_152259]	1.729	<0.001
TPX2	NM_012112	Homo sapiens TPX2, microtubule-associated, homolog (Xenopus laevis) (TPX2), mRNA [NM_012112]	1.725	<0.001
CDC25A	NM_001789	Homo sapiens cell division cycle 25 homolog A (S. cerevisiae) (CDC25A), transcript variant 1, mRNA [NM_001789]	1.724	<0.001
KIF2C	NM_006845	Homo sapiens kinesin family member 2C (KIF2C), mRNA [NM_006845]	1.723	<0.001
UBE2C	NM_181803	Homo sapiens ubiquitin-conjugating enzyme E2C (UBE2C), transcript variant 6, mRNA [NM_181803]	1.715	<0.001
ATAD2	NM_014109	Homo sapiens ATPase family, AAA domain containing 2 (ATAD2), mRNA [NM_014109]	1.709	<0.001
SPIRE1	NM_020148	Homo sapiens spire homolog 1 (Drosophila) (SPIRE1), mRNA [NM_020148]	1.707	<0.001
HMGA1	NM_145904	Homo sapiens high mobility group AT-hook 1 (HMGA1), transcript variant 6, mRNA [NM_145904]	1.7	<0.001
SPBC25	NM_020675	Homo sapiens spindle pole body component 25 homolog (S. cerevisiae) (SPBC25), mRNA [NM_020675]	1.698	<0.001
THC2530077	THC2530077	ALU5_HUMAN (P39192) Alu subfamily SC sequence contamination warning entry, partial (9%) [THC2530077]	1.698	<0.001
FOXM1	NM_202002	Homo sapiens forkhead box M1 (FOXM1), transcript variant 1, mRNA [NM_202002]	1.682	<0.001
MYBL2	NM_002466	Homo sapiens v-myb myeloblastosis viral oncogene homolog (avian)-like 2 (MYBL2), mRNA [NM_002466]	1.678	<0.001
CDCA8	NM_018101	Homo sapiens cell division cycle associated 8 (CDCA8), mRNA [NM_018101]	1.678	<0.001
NCAPG	NM_022346	Homo sapiens non-SMC condensin I complex, subunit G (NCAPG), mRNA [NM_022346]	1.677	<0.001
CDT1	NM_030928	Homo sapiens chromatin licensing and DNA replication factor 1 (CDT1), mRNA [NM_030928]	1.673	<0.001

E2F2	NM_004091	Homo sapiens E2F transcription factor 2 (E2F2), mRNA [NM_004091]	1.672	<0.001
KIFC1	NM_002263	Homo sapiens kinesin family member C1 (KIFC1), mRNA [NM_002263]	1.671	<0.001
CENPF	NM_016343	Homo sapiens centromere protein F, 350/400ka (mitosin) (CENPF), mRNA [NM_016343]	1.671	<0.001
CHEK1	NM_001274	Homo sapiens CHK1 checkpoint homolog (S. pombe) (CHEK1), mRNA [NM_001274]	1.665	<0.001
DEPDC1B	NM_018369	Homo sapiens DEP domain containing 1B (DEPDC1B), mRNA [NM_018369]	1.658	<0.001
DLG7	NM_014750	Homo sapiens discs, large homolog 7 (Drosophila) (DLG7), mRNA [NM_014750]	1.649	<0.001
CDT1	NM_030928	Homo sapiens chromatin licensing and DNA replication factor 1 (CDT1), mRNA [NM_030928]	1.645	<0.001
MCM10	NM_182751	Homo sapiens MCM10 minichromosome maintenance deficient 10 (S. cerevisiae) (MCM10), transcript variant 1, mRNA [NM_182751]	1.636	<0.001
TROAP	NM_005480	Homo sapiens trophinin associated protein (tastin) (TROAP), mRNA [NM_005480]	1.626	<0.001
MCM10	NM_182751	Homo sapiens MCM10 minichromosome maintenance deficient 10 (S. cerevisiae) (MCM10), transcript variant 1, mRNA [NM_182751]	1.622	<0.001
A_24_P324074	A_24_P324074	Unknown	1.618	<0.001
MELK	NM_014791	Homo sapiens maternal embryonic leucine zipper kinase (MELK), mRNA [NM_014791]	1.609	<0.001
CDCA5	NM_080668	Homo sapiens cell division cycle associated 5 (CDCA5), mRNA [NM_080668]	1.605	<0.001
CDCA3	NM_031299	Homo sapiens cell division cycle associated 3 (CDCA3), mRNA [NM_031299]	1.602	<0.001
ASPM	NM_018136	Homo sapiens asp (abnormal spindle) homolog, microcephaly associated (Drosophila) (ASPM), mRNA [NM_018136]	1.601	<0.001
CENPN	NM_018455	Homo sapiens centromere protein N (CENPN), mRNA [NM_018455]	1.6	<0.001
PTTG1	NM_004219	Homo sapiens pituitary tumor-transforming 1 (PTTG1), mRNA [NM_004219]	1.6	<0.001
A_24_P941540	A_24_P941540	Unknown	1.597	<0.001
LOC643228	XR_018267	PREDICTED: Homo sapiens similar to Glyceraldehyde-3-phosphate dehydrogenase (GAPDH) (LOC643228), mRNA [XR_018267]	1.596	<0.001
PKMYT1	NM_182687	Homo sapiens protein kinase, membrane associated tyrosine/threonine 1 (PKMYT1), transcript variant 2, mRNA [NM_182687]	1.595	<0.001
ADA	NM_000022	Homo sapiens adenosine deaminase (ADA), mRNA [NM_000022]	1.591	<0.001

HMMR	NM_012484	Homo sapiens hyaluronan-mediated motility receptor (RHAMM) (HMMR), transcript variant 1, mRNA [NM_012484]	1.587	<0.001
KIF11	NM_004523	Homo sapiens kinesin family member 11 (KIF11), mRNA [NM_004523]	1.586	<0.001
KIF15	NM_020242	Homo sapiens kinesin family member 15 (KIF15), mRNA [NM_020242]	1.585	<0.001
TACC3	NM_006342	Homo sapiens transforming, acidic coiled-coil containing protein 3 (TACC3), mRNA [NM_006342]	1.583	<0.001
UBE2S	NM_014501	Homo sapiens ubiquitin-conjugating enzyme E2S (UBE2S), mRNA [NM_014501]	1.577	<0.001
LOC644728	XR_018251	PREDICTED: Homo sapiens similar to Glyceraldehyde-3-phosphate dehydrogenase (GAPDH) (38 kDa BFA-dependent ADP-ribosylation substrate) (BARS-38) (LOC644728), mRNA [XR_018251]	1.573	<0.001
MND1	NM_032117	Homo sapiens meiotic nuclear divisions 1 homolog (S. cerevisiae) (MND1), mRNA [NM_032117]	1.562	<0.001
KNTC2	NM_006101	Homo sapiens kinetochore associated 2 (KNTC2), mRNA [NM_006101]	1.56	<0.001
EXO1	NM_003686	Homo sapiens exonuclease 1 (EXO1), transcript variant 3, mRNA [NM_003686]	1.559	<0.001
PTTG2	NM_006607	Homo sapiens pituitary tumor-transforming 2 (PTTG2), mRNA [NM_006607]	1.555	<0.001
NFKBIL2	NM_013432	Homo sapiens nuclear factor of kappa light polypeptide gene enhancer in B-cells inhibitor-like 2 (NFKBIL2), mRNA [NM_013432]	1.551	<0.001
PRC1	NM_003981	Homo sapiens protein regulator of cytokinesis 1 (PRC1), transcript variant 1, mRNA [NM_003981]	1.541	<0.001
DKFZp762E1312	NM_018410	Homo sapiens hypothetical protein DKFZp762E1312 (DKFZp762E1312), mRNA [NM_018410]	1.54	<0.001
CKAP2L	NM_152515	Homo sapiens cytoskeleton associated protein 2-like (CKAP2L), mRNA [NM_152515]	1.537	<0.001
LOC146909	ENST00000335534	Homo sapiens hypothetical protein LOC146909, mRNA (cDNA clone IMAGE:4418755), partial cds. [BC048263]	1.533	<0.001
ANLN	NM_018685	Homo sapiens anillin, actin binding protein (ANLN), mRNA [NM_018685]	1.526	<0.001
KIAA1794	NM_018193	Homo sapiens KIAA1794 (KIAA1794), mRNA [NM_018193]	1.525	<0.001
C18orf24	NM_001039535	Homo sapiens chromosome 18 open reading frame 24 (C18orf24), transcript variant 1, mRNA [NM_001039535]	1.522	<0.001
TNFRSF19L	NM_032871	Homo sapiens tumor necrosis factor receptor superfamily, member 19-like (TNFRSF19L), transcript variant 1, mRNA [NM_032871]	1.516	<0.001
DHFR	NM_000791	Homo sapiens dihydrofolate reductase (DHFR), mRNA [NM_000791]	1.513	<0.001
UBE2S	NM_014501	Homo sapiens ubiquitin-conjugating enzyme E2S (UBE2S), mRNA [NM_014501]	1.504	<0.001

GLT8D3	BC030023	Homo sapiens glycosyltransferase 8 domain containing 3, mRNA (cDNA clone MGC:33359 IMAGE:5266607), complete cds. [BC030023]	1.497	<0.001
GTSE1	NM_016426	Homo sapiens G-2 and S-phase expressed 1 (GTSE1), mRNA [NM_016426]	1.492	<0.001
C13orf3	BC013418	Homo sapiens chromosome 13 open reading frame 3, mRNA (cDNA clone MGC:4832 IMAGE:3604003), complete cds. [BC013418]	1.492	<0.001
SGOL1	NM_001012409	Homo sapiens shugoshin-like 1 (S. pombe) (SGOL1), transcript variant A1, mRNA [NM_001012409]	1.481	<0.001
PHF19	NM_001009936	Homo sapiens PHD finger protein 19 (PHF19), transcript variant 2, mRNA [NM_001009936]	1.478	<0.001
PLK1	NM_005030	Homo sapiens polo-like kinase 1 (Drosophila) (PLK1), mRNA [NM_005030]	1.476	<0.001
CENPE	NM_001813	Homo sapiens centromere protein E, 312kDa (CENPE), mRNA [NM_001813]	1.474	<0.001
KIF23	NM_138555	Homo sapiens kinesin family member 23 (KIF23), transcript variant 1, mRNA [NM_138555]	1.47	<0.001
LOC392454	XR_018314	PREDICTED: Homo sapiens similar to Proliferating cell nuclear antigen (PCNA) (Cyclin) (LOC392454), mRNA [XR_018314]	1.461	<0.001
BLM	NM_000057	Homo sapiens Bloom syndrome (BLM), mRNA [NM_000057]	1.449	<0.001
ASF1B	NM_018154	Homo sapiens ASF1 anti-silencing function 1 homolog B (S. cerevisiae) (ASF1B), mRNA [NM_018154]	1.441	<0.001
SEPT11	NM_018243	Homo sapiens septin 11 (SEPT11), mRNA [NM_018243]	1.439	<0.001
MTHFD2	NM_001040409	Homo sapiens methylenetetrahydrofolate dehydrogenase (NADP+ dependent) 2, methenyltetrahydrofolate cyclohydrolase (MTHFD2), nuclear gene encoding mitochondrial protein, transcript variant 2, mRNA [NM_001040409]	1.437	<0.001
LOC645256	XR_016875	PREDICTED: Homo sapiens similar to Glyceraldehyde-3-phosphate dehydrogenase (GAPDH) (LOC645256), mRNA [XR_016875]	1.435	<0.001
FEN1	NM_004111	Homo sapiens flap structure-specific endonuclease 1 (FEN1), mRNA [NM_004111]	1.43	<0.001
BG462058	BG462058	RST45053 Athersys RAGE Library Homo sapiens cDNA, mRNA sequence [BG462058]	1.428	<0.001
SHMT2	NM_005412	Homo sapiens serine hydroxymethyltransferase 2 (mitochondrial) (SHMT2), mRNA [NM_005412]	1.425	<0.001
KLHL7	BC009555	Homo sapiens kelch-like 7 (Drosophila), mRNA (cDNA clone IMAGE:3899090), complete cds. [BC009555]	1.425	<0.001
BOP1	NM_015201	Homo sapiens block of proliferation 1 (BOP1), mRNA [NM_015201]	1.423	<0.001
AURKB	NM_004217	Homo sapiens aurora kinase B (AURKB), mRNA [NM_004217]	1.42	<0.001

KIF22	NM_007317	Homo sapiens kinesin family member 22 (KIF22), mRNA [NM_007317]	1.419	<0.001
WDHD1	NM_007086	Homo sapiens WD repeat and HMG-box DNA binding protein 1 (WDHD1), transcript variant 1, mRNA [NM_007086]	1.417	<0.001
CEACAM20	ENST00000316962	Carcinoembryonic antigen-related cell adhesion molecule 20 precursor. [Source:Uniprot/SWISSPROT;Acc:Q6UY09] [ENST00000316962]	1.41	<0.001
TEAD4	NM_003213	Homo sapiens TEA domain family member 4 (TEAD4), transcript variant 1, mRNA [NM_003213]	1.408	<0.001
RNASEH2A	NM_006397	Homo sapiens ribonuclease H2, subunit A (RNASEH2A), mRNA [NM_006397]	1.405	<0.001
NCAPG2	NM_017760	Homo sapiens non-SMC condensin II complex, subunit G2 (NCAPG2), mRNA [NM_017760]	1.403	<0.001
A_24_P42071	A_24_P42071	Unknown	1.396	<0.001
FOXRED2	NM_024955	Homo sapiens FAD-dependent oxidoreductase domain containing 2 (FOXRED2), mRNA [NM_024955]	1.394	<0.001
NRAS	NM_002524	Homo sapiens neuroblastoma RAS viral (v-ras) oncogene homolog (NRAS), mRNA [NM_002524]	1.392	<0.001
MASTL	NM_032844	Homo sapiens microtubule associated serine/threonine kinase-like (MASTL), mRNA [NM_032844]	1.391	<0.001
LOC441572	XR_018368	PREDICTED: Homo sapiens similar to Glyceraldehyde-3-phosphate dehydrogenase (GAPDH) (LOC441572), mRNA [XR_018368]	1.39	<0.001
PCTK1	NM_033018	Homo sapiens PCTAIRE protein kinase 1 (PCTK1), transcript variant 2, mRNA [NM_033018]	1.387	<0.001
THC2549847	THC2549847	Unknown	1.386	<0.001
A_24_P75748	A_24_P75748	Unknown	1.378	<0.001
AI263083	AI263083	AI263083 qz35a02.x1 NCI_CGAP_Kid11 Homo sapiens cDNA clone IMAGE:2028842 3', mRNA sequence [AI263083]	1.374	<0.001
THC2520108	THC2520108	HSU09087 thymopoietin beta {Homo sapiens} (exp=-1; wgp=0; cg=0), partial (28%) [THC2520108]	1.363	<0.001
RFC4	NM_002916	Homo sapiens replication factor C (activator 1) 4, 37kDa (RFC4), transcript variant 1, mRNA [NM_002916]	1.362	<0.001
PFKL	NM_001002021	Homo sapiens phosphofructokinase, liver (PFKL), transcript variant 1, mRNA [NM_001002021]	1.361	<0.001
RNPS1	NM_006711	Homo sapiens RNA binding protein S1, serine-rich domain (RNPS1), transcript variant 1, mRNA [NM_006711]	1.358	<0.001

Genes overlapping with genelist published in Kalashnikova et al, Cancer Research 2010 marked in red

Supplementary Table S3 Drug signatures negatively correlated to ATAD2 high endometrial cancer

	**CMAP name**	**Description**	**N**	**P-value***
1	Puromycin	Antabiotics/Protein synthesis inhibitor	4	<0.0001
2	Alvespimycin	HSP-90 inhibitor	12	<0.0001
3	Trifluoperazine	Antipsychotic	16	<0.0001
4	Prochlorperazine	Dopamin receptor -antagonist	16	<0.0001
5	Thioridazine	Calmodulin-kinase inhibitor	20	<0.0001
6	Tanespimycin	HSP-90 inhibitor	62	<0.0001
7	LY-294002	PI3K inhibitor	61	<0.0001
8	Sirolimus	mTOR inhibitor	44	<0.0001
9	Trichostatin A	HDAC inhibitor	182	<0.0001
10	MS-275	HDAC inhibitor	2	<0.0001

N = number of instances in which the compounds were tested in the Connectivity map.

*The expression changes from the compounds tested were scored according to the ATAD2 expression signature and the *P* value for each compound represents the distribution of this score in the *N* instances as compared with the distribution of these scores among all compounds tested, using a permutation test.

Supplementary Table S4 GSEA external dataset from endometrial cancers*

Rank	Gene set Name- MSigDB C2	N	NES	P	FDR*
1	Shepard BMYB targets (Shepard *et al*, 2005)	57	2.83	0.0	0.0
2	Shepard crush and burn mutant DN (Shepard *et al*, 2005)	147	2.79	0.0	0.0
3	Dutertre estradiol response 24hr up (Dutertre *et al*, 2010)	242	2.78	0.0	0.0
4	Gavin FOXP3 targets cluster p6 (Gavin *et al*, 2007)	70	2.75	0.0	0.0
5	Odonnell TFRC targets DN (O'Donnell *et al*, 2006)	95	2.75	0.0	0.0

*Top 5 ranked gene sets with FDR<0.05 included

Supplementary Table S5:

Unique Patient ID	Tissue	Tissue	Met Loc
PrimMet_ID_101	Primary	Metastasis	Omental tissue
PrimMet_ID_102	Primary	Metastasis	Vulva
PrimMet_ID_103		Metastasis	Omental tissue
PrimMet_ID_104	Primary	Metastasis	Omental tissue
		Metastasis	Gastrointertinal tissue
PrimMet_ID_105	Primary	Metastasis	Ovarian tissue
PrimMet_ID_106	Primary	Metastasis	Spleen
PrimMet_ID_107	Primary	Metastasis	Parametrial tissue
PrimMet_ID_108		Metastasis	Abdominal tissue
PrimMet_ID_109	Primary	Metastasis	Isthmus/Cervix
PrimMet_ID_110	Primary	Metastasis	Douglasi
		Metastasis	Lymph node
		Metastasis	Lymph node
PrimMet_ID_111	Primary	Metastasis	Lymph node
		Metastasis	Lymph node
		Metastasis	Lymph node
PrimMet_ID_112	Primary	Metastasis	Cervical tissue
PrimMet_ID_113	Primary	Metastasis	Cervical tissue
		Metastasis	Vaginal tissue
PrimMet_ID_114	Primary	Metastasis	Ovarian tissue
		Metastasis	Ovarian tissue
		Metastasis	Vaginal tissue
PrimMet_ID_115	Primary	Metastasis	Vaginal tissue
PrimMet_ID_116	Primary	Metastasis	Vaginal tissue
PrimMet_ID_117	Primary	Metastasis	Vaginal tissue
PrimMet_ID_118	Primary	Metastasis	Uterine wall
		Metastasis	Lymph node
PrimMet_ID_119		Metastasis	Vaginal tissue
PrimMet_ID_120	Primary	Metastasis	Ovarian tissue
PrimMet_ID_121	Primary	Metastasis	Lymph node
		Metastasis	Lymph node
PrimMet_ID_122	Primary	Metastasis	Isthmus/Cervix
PrimMet_ID_123	Primary	Metastasis	Ovarian tissue
PrimMet_ID_124	Primary	Metastasis	Abdominal tissue
PrimMet_ID_125	Primary	Metastasis	Vaginal tissue
PrimMet_ID_126	Primary	Metastasis	Vaginal tissue
PrimMet_ID_127	Primary	Metastasis	Vaginal tissue
PrimMet_ID_128		Metastasis	Abdominal tissue
		Metastasis	Abdominal tissue
PrimMet_ID_129	Primary	Metastasis	Cervical tissue
PrimMet_ID_130	Primary	Metastasis	Omental tissue
PrimMet_ID_131		Metastasis	Vaginal tissue

Primary	Corresponding primary tumor
Metastasis	Metastases with corresponding primary tumor
Metastasis	Metastases without corresponding primary tumor
